# Munc18‐2 is required for Syntaxin 11 Localization on the Plasma Membrane in Cytotoxic T‐Lymphocytes

**DOI:** 10.1111/tra.12337

**Published:** 2015-11-02

**Authors:** Nele M.G. Dieckmann, Yvonne Hackmann, Maurizio Aricò, Gillian M. Griffiths

**Affiliations:** ^1^ Cambridge Institute for Medical Research, Cambridge Biomedical CampusUniversity of CambridgeCambridgeCB2 0XYUnited Kingdom; ^2^ Current address: Biochemistry CenterHeidelberg UniversityHeidelbergGermany; ^3^ Azienda Sanitaria Provinciale 7Piazza Igea 1I‐97100RagusaItaly

**Keywords:** CTL, FHL4, FHL5, Munc18‐2, Syntaxin 11

## Abstract

Cytotoxic T‐lymphocytes (CTL) kill their targets by cytolytic granule secretion at the immunological synapse. The Sec/Munc protein, Munc18‐2, and its binding partner Syntaxin 11 (STX11) are both required for granule secretion, with mutations in either leading to the primary immunodeficiency, Familial Haemophagocytic Lymphohistiocytosis (FHL4 and 5). Understanding how Munc18‐2 and STX11 function in CTL has been hampered by not knowing the endogenous localization of these proteins. Using a novel FHL5 Munc18‐2 mutation that results in loss of protein, cytotoxicity and degranulation together with CTL from an FHL4 patient lacking STX11, enabled us to localize endogenous STX11 and Munc18‐2 in CTL. Munc18‐2 localized predominantly to cytolytic granules with low levels associated with the plasma membrane where STX11 localized. Importantly, while Munc18‐2 localization is unaffected by the absence of STX11 in FHL4 CTL, STX11 is lost from the plasma membrane in FHL5 CTL lacking Munc18‐2. These findings support a role for Munc18‐2 in chaperoning STX11 to the plasma membrane where the final fusion events involved in secretion occur.

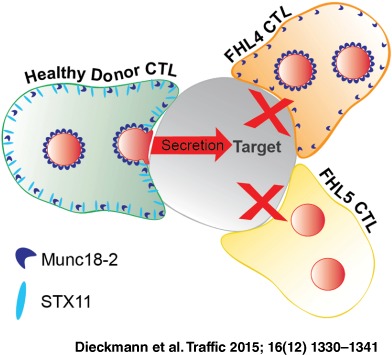

CD8‐positive cytotoxic T‐lymphocytes (CTL) are key players of the mammalian immune system, providing protection from viral infection and cancerous cells. CTL kill through precise delivery of perforin and granzymes across a highly organized CTL:target interface that is known as the immune synapse. CTL protect themselves from the potent pro‐apoptotic factors by storing these proteins in specialized secretory lysosomes, termed cytolytic granules. During CTL attack, these granules are delivered to the synapse where they fuse with the plasma membrane and release their deadly contents onto the target (reviewed in 1).

The biogenesis of cytolytic granules and their final fusion with the plasma membrane requires a specialized fusion machinery. Two components of this are the **SNA**P (Soluble NSF Attachment Protein) **Re**ceptor (SNARE)‐protein Syntaxin 11 (STX11) 2 and the Syntaxin binding protein Munc18‐2 3, 4 and genetic mutations that cause loss of function of either of these proteins gives rise to Familial Haemophagocytic Lymphohistiocytosis (FHL), a devastating autosomal hereditary immune deficiency condition that is marked by the failure of CTL to kill infected cells efficiently (reviewed by 5 6).

In neuronal and epithelial cells, Munc18 proteins were found to be required for stable plasma membrane localization of Syntaxin 1A (STX1A) 7, 8. Interestingly, in FHL5 patient CTL (in which Munc18‐2 is lost) STX11 levels are usually strongly reduced and the inability of STX11 and Munc18‐2 deficient cells to kill target cells efficiently was shown to be due to a degranulation defect 3, 4, 9. This led to the suggestion that a chaperoning interaction similar to the Munc18/STX1A pair is given between Munc18‐2 and STX11 and that the two proteins drive the last step of granule secretion at the CTL plasma membrane 3, 4, 9, 10, 11, 12. Notably, the finding that FHL5 patients with mutations R65Q/W retain the ability to interact with STX11 supports an additional direct role in secretion for Munc18‐2 13. Whether Munc18‐2 and STX11 function at the plasma membrane, or at a late granule maturation step 14 remains unclear, in part due to the lack of information on the precise localization of these proteins.

STX11 and Munc18‐2 are widely expressed 15, 16, 17 and studies in other secretory cells flagged up several roles for Munc18‐2 on compartments other than the plasma membrane. In mast cells, Munc18‐2 on the limiting membrane of secretory granules appeared to provide a link to microtubules and thus promote the transport of granules to the cell periphery 18. A study in the pancreatic beta‐cell line MIN6 reported a role for Munc18‐2 in modulating calcium responsiveness during the secretion of granules. Sucrose gradient analysis detected Munc18‐2 predominantly in low‐density fractions that correspond to ‘small membranous organelles and soluble proteins’ 19. Furthermore, it has been suggested that in CTL the granules themselves undergo one (or several) maturation steps before they become ‘secretion competent bullets’ that carry all the machinery required for fusion at the plasma membrane 14. These reports highlight multiple potential roles for Munc18‐2 but exactly when and where STX11 and Munc18‐2 function in CTL is not fully understood.

Precise localization of proteins within the cellular environment can give important clues about their function. However, several issues have been reported previously when it comes to the localization of tagged SNARE‐protein and Munc18‐2 constructs: One of the key functional domains of any SNARE‐protein is either a transmembrane domain or a lipidic membrane anchor enabling these proteins to associate directly with membranes (review: 20 21). Thus, when a SNARE is even slightly over‐expressed, the possibility of mis‐localization of the tagged construct to non‐physiological compartments is a major concern. Although it has been reported that over‐expressed STX11 localizes to the recycling endosome in human CTL it is not known whether this corresponds to localization of the endogenous protein 12, 22. Munc18‐2 on the other hand has no domains that suggest an ability to associate with membranes independently of helper proteins. Indeed over‐expressed Munc18‐2 constructs remain diffuse cytoplasmic 23 unless a membrane‐associated binding partner such as STX11 is expressed in parallel 24.

We therefore assessed the distribution of endogenous Munc18‐2 and STX11 in CTL. Four new polyclonal antibodies were raised against near full‐length human Munc18‐2 and STX11 protein. By comparing immunofluorescence microscopy (IF) in healthy donor (HD) CTL versus FHL4 and FHL5 patient cells, lacking Munc18‐2 or STX11, we were able to determine specific staining and now show the localization of endogenous Munc18‐2 and STX11 in human CTL. In order to do this we also report a novel patient mutation in Munc18‐2 that abolishes protein expression, and used CTL from a previously reported patient mutation lacking STX11. This approach allowed us to define the endogenous localization of both proteins. Our data reveal that endogenous Munc18‐2 localizes predominantly to the cytolytic granules, with low levels on the plasma membrane, and is required for STX11 delivery to the plasma membrane.

## Results

### FHL4 and FHL5 CTL validate STX11 and Munc18‐2 antibody specificity

**Figure 1:**
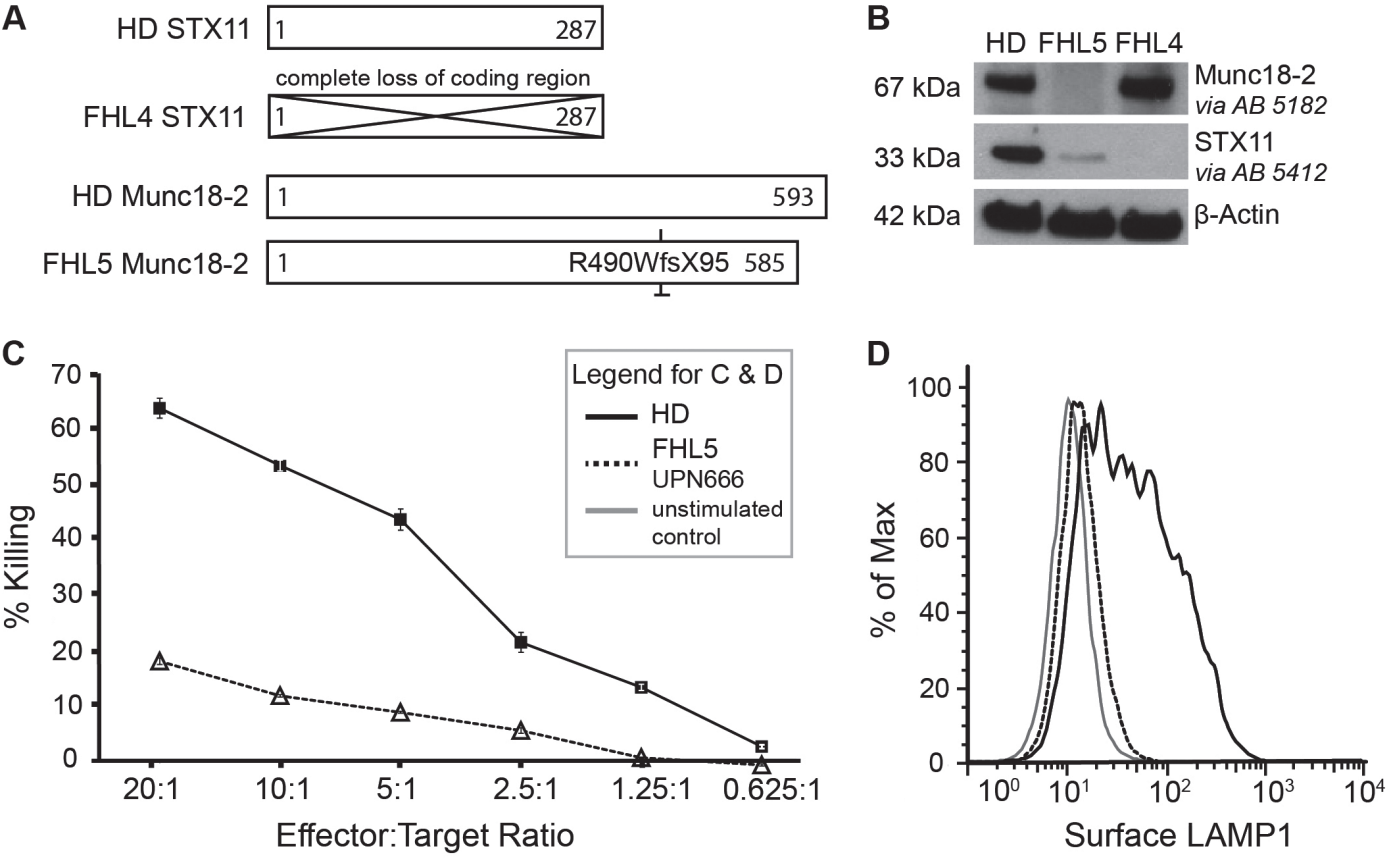
**FHL4 and FHL5 patient CTL establish Syntaxin 11 and Munc18‐2 antibody specificity.** A) Schematic representation of the STX11 and Munc18‐2 amino acid sequences indicating the effects of the homozygous mutations present in the FHL4 (847–1850) and FHL5 (UPN666) patient cells used in this study compared to healthy donor (HD). B) Western Blots on human CTL lysates against Munc18‐2 and STX11 using the two polyclonal rabbit antibodies 5182 and 5412. C) Percentage of P815 target cells lysis (% killing) for HD (solid black line, squares) or FHL5 patient UPN666 CTL (dotted black line, triangles) at Effector:Target ratios shown. CTL,10 days post restimulation. D) Granule release assay showing surface LAMP1 after 30 minutes PMA‐Ionomycin stimulation of CTL from HD (solid black) and FHL5 patient UPN666 (dotted black line). Solid gray line: unstimulated HD CTL. CTL, 10 days post restimulation.

FHL4 CTL with a homozygous loss of the entire STX11 coding exon (AL135917:g.25561–44749_del, 25) and FHL5 CTL with a homozygous deletion‐insertion mutation (c.1468_1470delCGGinsTGGACAGCCCTGGACAGGG p.R490WfsX95, UPN666), leading to a frameshift from amino acid 490 onwards and predicting a premature stop‐codon were used to confirm the antibody specificities (Figure 1A).

Western blotting with the Munc18‐2 and STX11 antibodies 5182 and 5412 showed a complete loss of Munc18‐2 and STX11 protein from the FHL4 and 5 CTL, respectively. As described previously for other FHL5 patients 3, 4 STX11 levels are reduced in the absence of Munc18‐2 (Figure 1B).

The frameshift mutation in UPN666 has not been described before. We therefore investigated the effects of this novel mutation on CTL killing and degranulation and found both to be reduced even after 2 weeks of culture in the presence of IL‐2 (Figure 1C,D). This stands in contrast to several previously published FHL5 patient CTL that did not show abnormal cytotoxicity in culture 26.

### Endogenous Munc18‐2 localizes to cytolytic granules and the plasma membrane in human CTL

**Figure 2:**
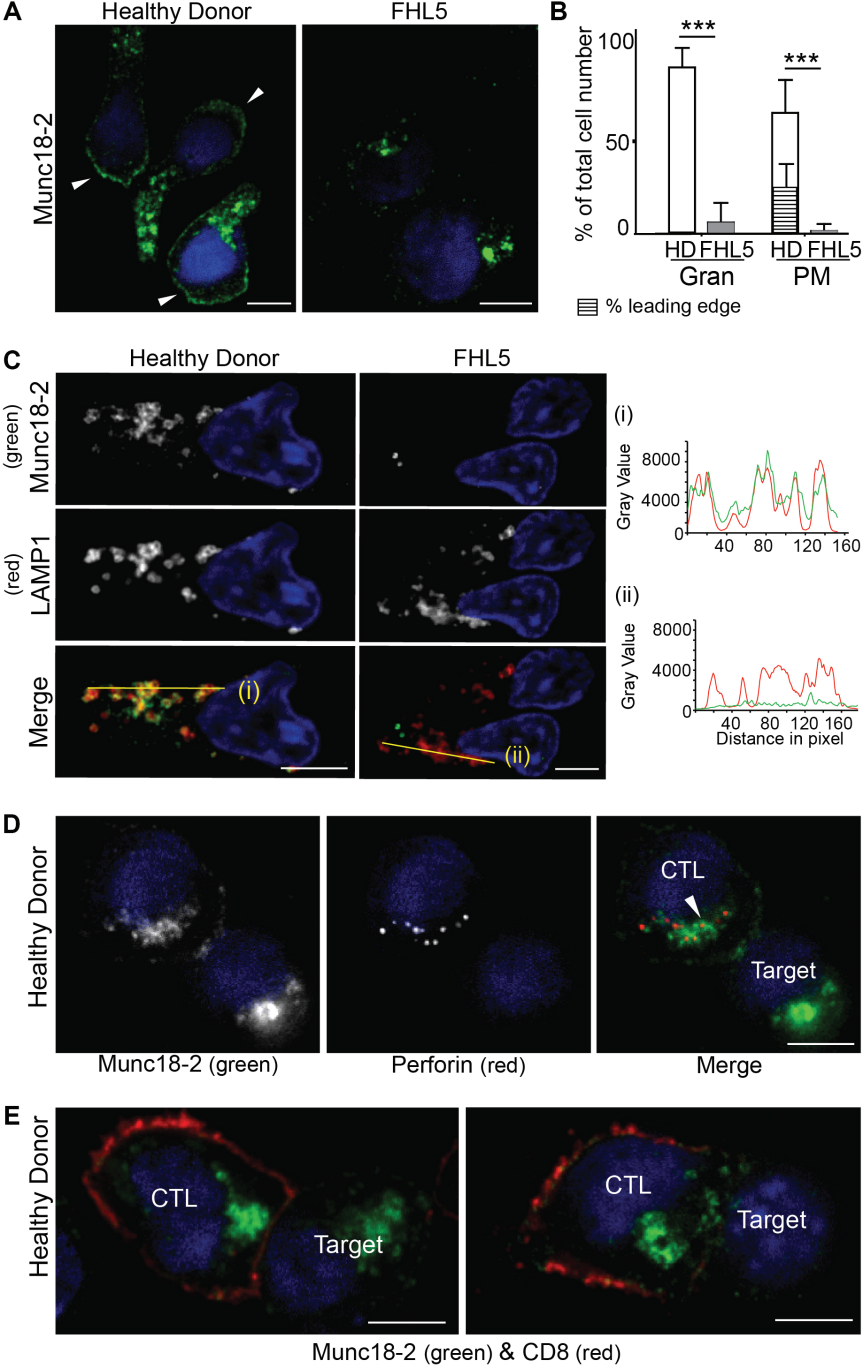
**Endogenous Munc18‐2 localizes predominantly to cytolytic granules in human CTL.** A) Immunofluorescence microscopy showing localization of Munc18‐2 (antibody 5182) in healthy donor (HD) and FHL5 CTL (UPN666). White arrowheads: leading edge. B) Quantitation of Munc18‐2 antibody staining from 6 independent experiments: HD (white, n = 397) and FHL5 (gray, n = 374). Gran: granular staining, PM: plasma membrane staining, hatched bar = HD CTL with an accumulation of Munc18‐2 at the leading edge. Shown is the mean with standard deviation, *** indicates a significant difference with p < 0.0001, two‐tailed students t‐test (unpaired), 95% confidence limits. C) Co‐staining for Munc18‐2 (green) and LAMP1 (red) in three independent experiments comparing HD and FHL5 CTL (n = 158 each). Yellow lines (i) and (ii) were drawn via the imageJ line tool (linewidth: 3 pixel) to obtain Munc18‐2 and LAMP1 intensity profiles. Imaris display adjustments optimized for granular signal. D) Co‐staining of a CTL‐P815 conjugate for Munc18‐2 (green) and perforin (red, exclusively expressed in CTL). The white arrowhead indicates a perforin core surrounded by Munc18‐2 (n = 119 HD, 2 independent experiments). E) Co‐staining of a CTL‐P815 conjugate for Munc18‐2 (green) and CD8 (red) showing polarization of granule‐associated Munc18‐2 toward the synapse (n = 63). All images show single confocal planes. Scale bars: 5 µm.

We tested both anti‐Munc18‐2 antibodies (5182 and 5184) by IF on HD as well as FHL5 CTL (Figure 2). In HD derived CTL, both antibodies localized predominantly to intracellular granules and a central puncta, with low levels detected on the plasma membrane. We subsequently focused on antibody 5182 because it provided a stronger signal. By staining HD and FHL5 patient cells in parallel we confirmed that the granular and the plasma membrane staining are specific to Munc18‐2 (Figure 2A,B). Colocalization with LAMP1 (*n* = 158 HD cells) and the cytolytic granule marker perforin (*n* = 119 HD cells) identified the predominant granular staining as the cytolytic granules. Munc18‐2 colocalized with LAMP1 on the limiting membrane of the granule (Figure 2C) surrounding perforin (Figure 2D). This staining was lost from FHL5 CTL (Figure 2A–D) while the central puncta, identified as the centrosome by gamma‐tubulin colocalization, remained (data not shown). A weak cytoplasmic staining could also be detected in HD cells, consistent with a small pool of Munc18‐2 dispersed throughout the cytoplasm. With six independent experiments revealing strong granular staining in HD CTL, our experiments suggest that endogenous Munc18‐2 is localized to cytolytic granules in CTL. Although some plasma membrane staining was observed it was weaker and variable between experiments (Figure 2A–D). When Munc18‐2 was observed on the plasma membrane an accumulation was seen at the leading edge in 30% of cells (Figure 2A,B).

As Munc18‐2 localized to cytolytic granules in CTL, we asked whether this was maintained when granules polarized toward target cells. Two‐thirds of CTL‐target conjugates showed granules clustered at the immunological synapse, and in each case Munc18‐2 remained associated with the granules (Figure 2E, n = 63). Any plasma membrane staining, remained equally distributed in all conjugates without accumulation at the synapse (Figure 2E).

### Endogenous STX11 associates with the plasma membrane accumulating at the leading edge of human CTL

We next sought to determine whether STX11 localized to the same cellular compartments as Munc18‐2. Again, both our custom‐made STX11 antibodies (5412 and 5413) showed the same staining pattern in CTL and one antibody (5412) yielded considerably stronger signal.

**Figure 3:**
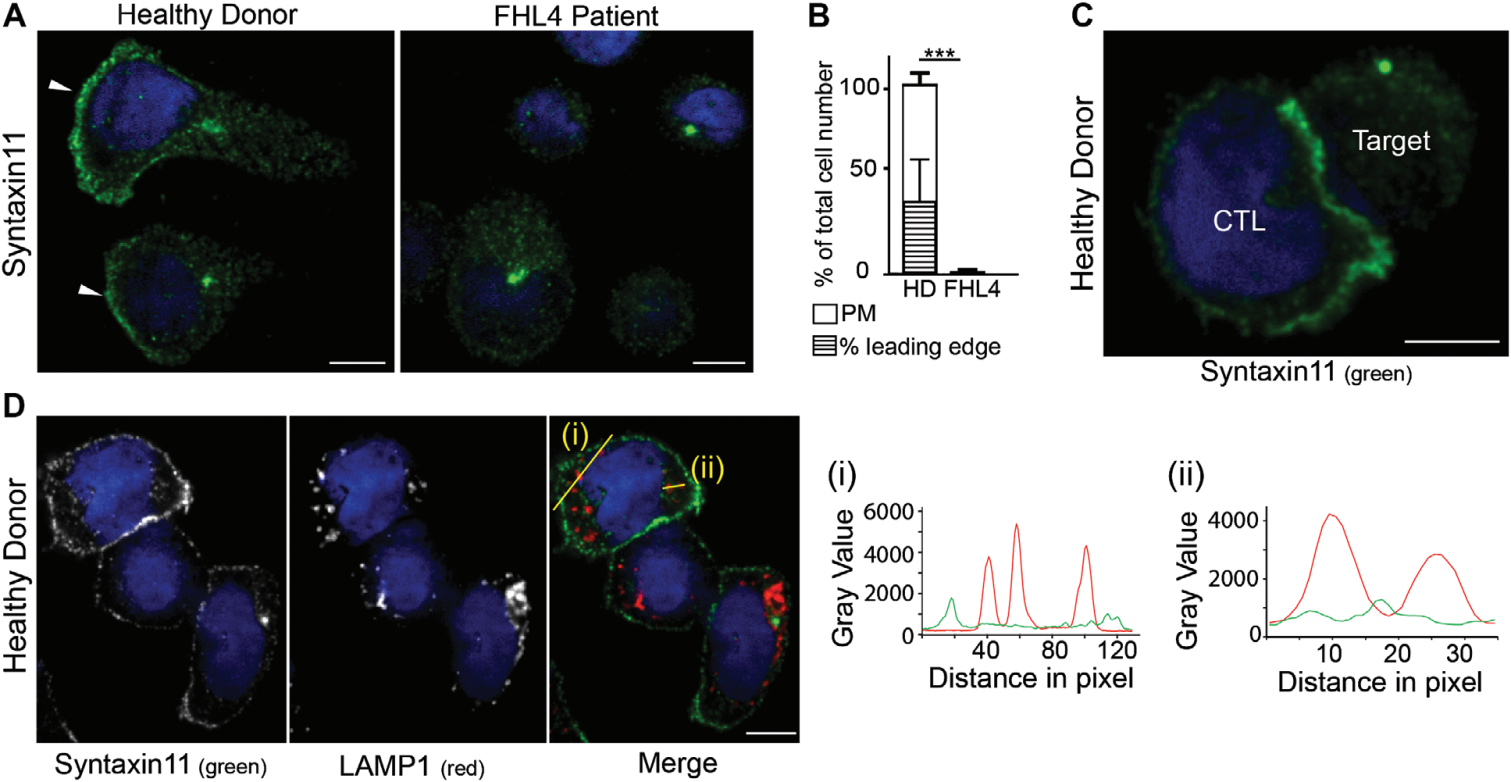
**Endogenous STX11 is on the plasma membrane of human CTL.** A) Localization of STX11 in healthy donor (HD) and FHL4 patient 847–1850 CTL using antibody 5412. White arrowheads: leading edge. B) Percentage of cells with plasma membrane staining for HD (white, n = 328) and FHL4 (black, n = 312) CTL using antibody 5412. PM: plasma membrane staining, hatched bar = HD CTL with an accumulation of STX11 at the leading edge. Results shown as mean from five independent experiments with standard deviation, *** indicate a significant difference with p < =0.0001, two‐tailed students t‐test (unpaired), 95% confidence limits. C) STX11 staining using antibody 5412 in HD CTL‐target conjugates illustrating STX11 accumulation at the synapse (5 experiments, n = 128). D) Co‐staining for anti‐STX11 5412 (green) and LAMP1 (red) in HD cells (2 experiments, n = 135). Yellow lines (i) and (ii) were drawn via the imageJ line tool (linewidth: 3 pixel) to obtain STX11 and LAMP1 intensity profiles. All images show single confocal planes. Scale bars: 5 µm.

In five independent experiments we compared STX11 staining with 5412 in HD cells (n = 328) and FHL4 control cells (n = 312). We observed staining of the plasma membrane exclusively in HD cells while punctate cytoplasmic and centrosome staining was found in both HD and FHL4 cells, suggesting that these signals were non‐specific (Figure 3A). It should be noted that as cells were fixed with methanol any free cytoplasmic pool of STX11 would be lost after fixation. Hundred percent of HD CTL showed plasma membrane localization of STX11, with 30% showing accumulation at the leading edge (Figure 3B). In CTL‐target conjugates, STX11 accumulated either across or clustered at the immune synapse in ≤16% of conjugates (Figure 3C). In contrast to Munc18‐2 we did not observe colocalization of STX11 with LAMP1 (Figure 3D).

### Munc18‐2 localization is independent of STX11 but in the absence of Munc18‐2, STX11 is lost from the plasma membrane

It has been suggested that Munc18‐2 might act as a chaperone to transport STX11 to the plasma membrane as observed for the Munc18/STX1A pair 7, 8. Therefore, we asked whether the localization of endogenous STX11 at the plasma membrane depends upon the presence of Munc18‐2 and vice versa. We performed parallel Munc18‐2 and STX11 staining in HD, FHL4 and FHL5 cells in three independent experiments and co‐stained with LAMP1 or CD8 in order to assess potential effects on colocalization with these markers.

**Figure 4:**
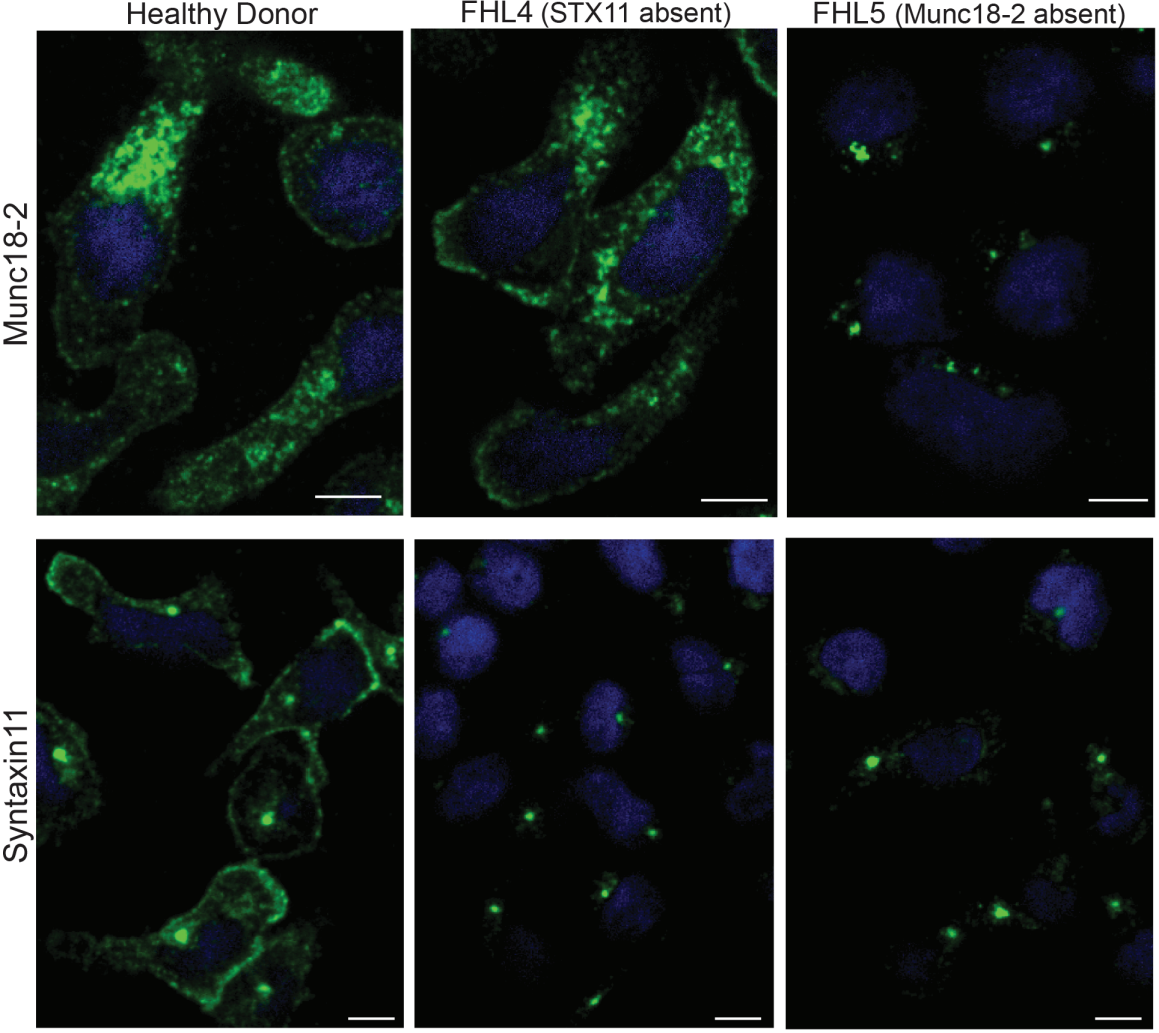
**Munc18‐2 localization is independent of STX11 but in the absence of Munc18‐2, STX11 is lost from the plasma membrane**. Localization of endogenous Munc18‐2 and STX11 in CTL from healthy donor (HD) (n = 265), FHL4 (847–1850, n = 252) and FHL5 (UPN666, n = 263) using antibodies 5182 and 5412 respectively. All images show single confocal planes and represent three independent experiments. Scale bars: 5 µm.

**Figure 5:**
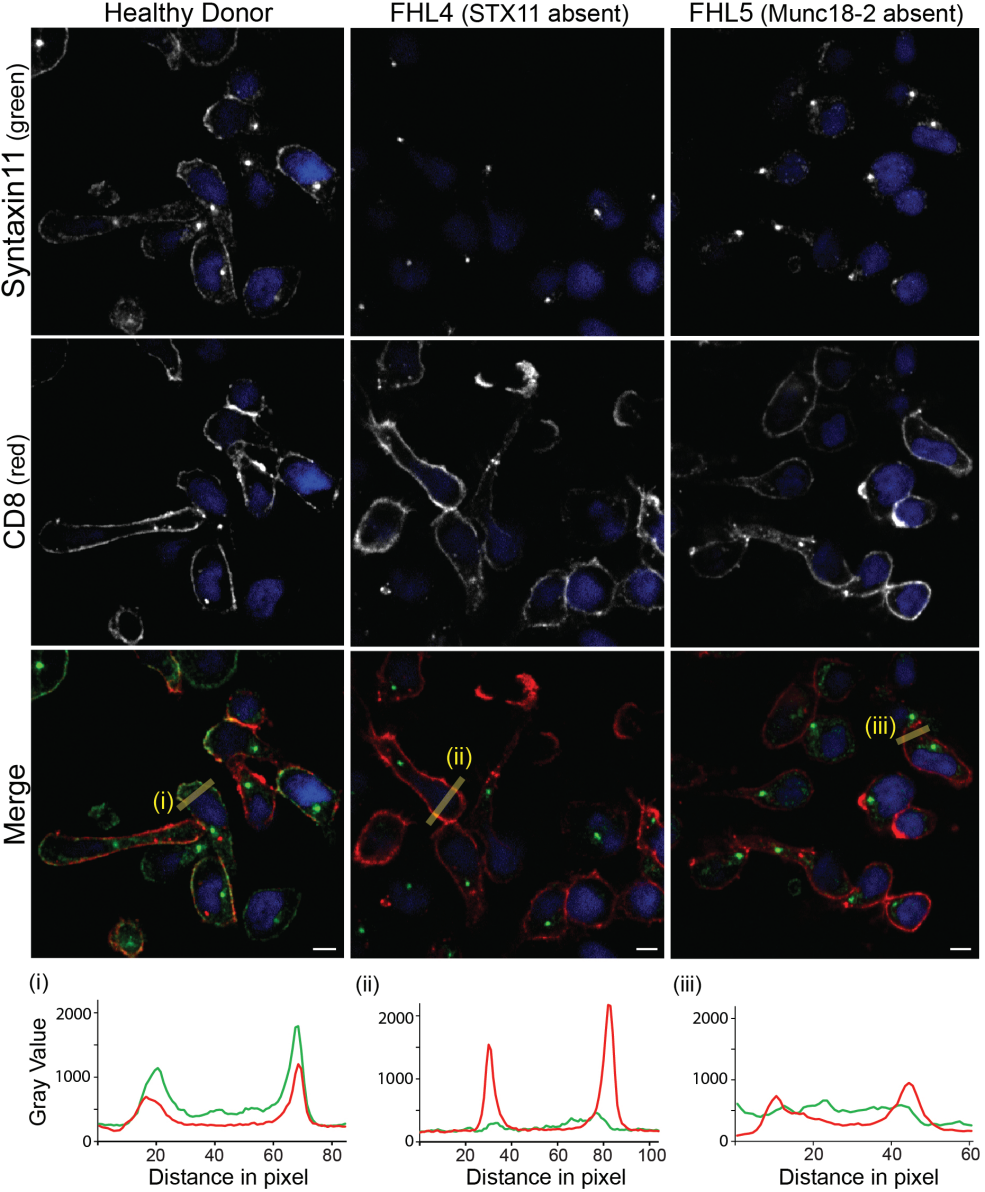
**STX11 colocalizes with CD8 at the plasma membrane.** Co‐staining for anti‐STX11 5412 (green) and plasma membrane marker CD8 (red) in healthy donor (HD) (n = 149, 4 experiments), FHL4 (847–1850, n = 144, 4 experiments) and FHL5 (UPN666, n = 56, 1 experiment) CTL. Yellow lines (i) and (ii) were drawn via the imageJ line tool (linewidth: 15 pixel) to obtain STX11 and CD8 intensity profiles. All images show single confocal planes. Scale bars: 5 µm.

As observed previously, we found strong Munc18‐2 staining on granules and weaker staining on the plasma membrane in HD cells (*n* = 265) but no Munc18‐2 staining on FHL5 cells (*n* = 263). Centrosomal staining was non‐specific as it was present in both HD and patient cells. Munc18‐2 staining in FHL4 cells (*n* = 252) resembled staining in HD cells, indicating that Munc18‐2 localization was not affected by the absence of STX11. In contrast, FHL5 CTL (lacking Munc18‐2) completely lost the plasma membrane STX11 localization seen in HD CTL (Figure 4), confirmed by colocalization with the T cell plasma membrane protein, CD8 (Figure 5). These results support a role for Munc18‐2 in STX11 stability and are consistent with a role for Munc18‐2 in chaperoning STX11 to the plasma membrane.

## Discussion

Munc18‐2 and STX11 play essential roles in secretion from CTL and NK cells. Mutations in the genes encoding these two proteins give rise to FHL, a profound immunodeficiency that can be cured only by bone marrow transplantation. Functional studies have shown that these proteins play a role in secretion 2, 3, 4, but do not reveal at which stage in the secretory pathway they are required. In order to identify the sites of action of Munc18‐2 and STX11 we asked where the endogenous proteins were localized in CTL.

There are two well‐acknowledged problems with establishing the cellular localization of SNARE and associated Munc18 proteins: over‐expression often results in mis‐localization because SNARE component proteins can recombine promiscuously and levels of binding partners become limiting 7, 20, 27. Furthermore, the multitude of homologous proteins (15 syntaxins; 3 Munc18‐proteins) means that it can be difficult to determine the specificity of antibody binding for any single protein. In order to overcome these difficulties, we investigated the localization of endogenous STX11 and Munc18‐2 in human CTL through the use of novel antibodies raised against (near) full‐length human Munc18‐2 and STX11 protein. Importantly, the use of patient CTL lacking STX11 (FHL4) and Munc18‐2 (FHL5) allowed us to establish the specificity of staining. Our results demonstrate that endogenous Munc18‐2 is localized predominantly on secretory lysosomes of CTL although lower levels are observed on the plasma membrane, where STX11 is found, raising the possibility that Munc18‐2 might transit the plasma membrane and deliver STX11 to this site.

Previous studies in CTL and NK cells have examined the localization of over‐expressed, tagged constructs of these proteins 11, 12, 22, 24, 28 or using antibodies raised against peptide or mouse epitopes in human CTL 13, 29. These studies suggested many possible localizations in CTL, with Munc18‐2 diffusely cytoplasmic 24, on dense puncta 13 or associated with cytolytic granules 24 and STX11 on distinct cytoplasmic puncta 11, 12, 22, 28, 29 colocalizing with CD3 and granzyme B 12 but not perforin 28, 29 and/or associated with the plasma membrane 12, 24.

Our localization of endogenous STX11 staining confirms the plasma membrane component of the localization observed when TFP‐STX11 and GFP‐STX11 was expressed in human CTL and YTS NK cells, respectively 12, 24. However, we do not observe the additional localization of STX11 on vesicular structures observed when STX11 is over‐expressed 12, 22, 24, 28 or with previous antibody localizations 13, 29. Although vesicular staining was observed in several studies, there was little consensus over the identity of this compartment. Recent reports based on over‐expressed TFP‐STX11 colocalization with mCherry‐Rab11a propose a model by which STX11 is only delivered to the plasma membrane immediately prior to granule fusion via VAMP8‐mediated fusion of the recycling endosome 12, 22. In the present study, we show that there is a steady‐state localization of endogenous STX11 on the plasma membrane, which requires Munc18‐2 for its delivery to this site.

Interestingly in a previous study the diffuse cytoplasmic GFP‐Munc18‐2 localization changed to a vesicular colocalization with mcherry‐Munc18‐2 when both Munc18‐2 and STX11 were over‐expressed, demonstrating the inter‐dependence of these two proteins and the importance of determining the endogenous localization of these proteins with physiological expression levels 24.

The localization of Munc18‐2 on cytolytic granules in CTL that we observe matches the endogenous Munc18‐2 staining observed on the secretory granules of mast cells 18, 30 and in neutrophils 31. Intriguingly these granules are all secretory lysosomes, suggesting that Munc18‐2 localizes to this compartment in immune cells.

We found the distribution of Munc18‐2 to be the same in HD cells and STX11‐deficient FHL4‐cells. This suggests that Munc18‐2 does not depend on STX11 for its association with granule membranes and the plasma membrane and could be explained by Munc18‐2 associating with an alternative SNARE‐protein in the absence of STX11. Hackmann et al. 25 demonstrated that the N‐terminal peptide of the STX11 homologue Syntaxin3 (STX3) binds Munc18‐2 in vitro (albeit with 20 times lower affinity compared to the STX11 peptide) and that STX3 expression is upregulated through IL‐2 culture of human NK cells, a culture condition that has previously been reported to partially restore granule release and killing through FHL4 and five NK cells and CTL 2, 3, 4, 32. Hackmann et al. furthermore showed STX3 re‐localized from intracellular vesicles to the plasma membrane in STX11 deficient CTL. These findings suggest STX3 as one likely candidate for interaction with Munc18‐2 at the granule and plasma membrane in the absence of STX11. Conversely, STX11 is lost from the plasma membrane in FHL5 patient cells lacking Munc18‐2. STX11 thus appears to require Munc18‐2 either to reach the plasma membrane or to remain stably associated with it, although we cannot rule out the possibility that Munc18‐2 might stabilize STX11 at an earlier stage during its biosynthesis. Either way, Munc18‐2 appears to be required to build up a steady‐state pool of STX11 on the plasma membrane of CTL.

Two observations invite further speculation on additional roles for both proteins: STX11 readily accumulates at the leading edge of migrating CTL but we only rarely observed an accumulation at the immune synapse. Fusion of the small number of cytolytic granules that is needed to eliminate a target may not necessarily require a high density of SNARE proteins. At the leading edge of a migrating cell, however, a high number of vesicles that deliver migratory or target sensing factors to the plasma membrane may need to be turned over 33 and it is tempting to speculate that STX11 may be involved in these processes. Similarly, based on the fact that only Munc18‐2 (but not STX11) localized to CTL granules it is possible that Munc18‐2 may play additional roles on this compartment as previously suggested for granular Munc18‐2 in mast cells 18.

## Materials and Methods

### Cells

Patient UPN666 presented at the age of 1 month with full‐blown HLH (fever, splenomegaly, bi‐cytopenia, low plasma levels of fibrinogen, high levels of triglycerides and ferritin). Functional study showed defective GRA results; sequencing of the Syntaxin binding protein 2 (Munc18‐2) (*STXBP2*) gene revealed a homozygous deletion‐insertion mutation (c.1468_1470delCGGinsTGGACAGCCCTGGACAGGG p.R490WfsX95), leading to a frameshift at amino acid 490 and predicting a premature stop‐codon 95 amino acids later; thus, FHL5 was diagnosed. Ethical approval was obtained in accordance with the standards of the Declaration of Helsinki. A bulk CD8 line (CTL) FHL4 patient (847–1850) was described previously 25. Bulk CD8+ cells from the FHL5 patient (UPN666) were isolated by negative selection from peripheral blood mononuclear cells (PBMCs) using the Dynabeads Untouched Human CD8 T‐cell purification kit from Invitrogen. HD CTL were from donor 434–722.

### Cell culture

CTL were cultured at 37°C in a humidified atmosphere with 8–10% CO_2_ in RPMI 1640, 2 mm l‐glutamine, 1 mm sodium pyruvate, 0.9 m sodium bicarbonate, 50 µm beta‐mercaptoethanol (Invitrogen‐GIBCO), 5% human AB‐Serum (SeraLab) and recombinant human IL‐2 (∼100 U/mL). CTL were stimulated every 3–4 weeks using irradiated PBMCs isolated from buffy coats in the presence of PHA. Whole human blood was collected from HDs with informed consent from individuals and prevented from coagulation by addition of sterile sodium citrate. PBMCs were isolated by Ficoll‐Paque™ (GE Healthcare) gradient centrifugation, irradiated at 3000 radiation absorbed dose (rad) of gamma rays and seeded at a 1:1 ratio with human CTL in a 24‐well plate and a final concentration of 1 µg/mL PHA (Roche). CTL were imaged 10–15 days post stimulation, when maximal killing capacity was reached. Purity of CTL populations (80–99%) was confirmed by flow cytometry with anti‐human CD8‐allophycocyanin (APC) [MEM‐31] and anti‐human CD4‐R‐phycoerythrin (PE) [MEM‐241] antibodies (both Abcam) prior to use.

### Antibodies and reagents

Polyclonal rabbit antibodies were raised against full‐length human Munc18‐2 (5182 and 5184) and STX11ΔCys‐GST (5412 and 5413) as previously described 25. Other antibodies were sourced: mouse anti‐human LAMP1 [H4A3] (Iowa University Hybridoma cell bank), mouse anti‐human CD8 [UCHT4] (Sigma‐Aldrich, C7423), mouse anti‐human perforin [δG9] (BD Pharmingen, 556434), mouse anti‐beta actin [AC‐15] (Sigma‐Aldrich, A5441), mouse anti‐human LAMP1‐PE [H4A3] (eBioscience, 12–1079), mouse anti‐human CD8‐APC [MEM‐31] (Abcam, ab26004), mouse anti‐human CD4‐PE [MEM‐241] (Abcam, ab18282). Secondary antibodies goat anti‐rabbit‐AF488 (A11034, highly cross‐adsorbed) and goat anti‐mouse‐AF568 (A11031, highly cross‐adsorbed) and Hoechst nuclear stain 33342 were from Invitrogen. HRP‐conjugated secondary antibodies used for immunoblotting were goat anti‐rabbit IgG (Jackson, 111‐035‐144) and goat anti‐mouse IgG (Jackson, 113‐035‐146). Ionomycin and PMA were obtained from Sigma‐Aldrich.

### Immunoblotting

CTL were washed 2–3× in ice‐cold sterile PBS. Cells (4 × 10^7^/mL) were lysed in lysis buffer (50 mm Tris–HCl, 100 mm NaCl, 5 mm Mg Cl_2_, 1% Triton X‐100, pH 7.6) supplemented with Protease Inhibitor Cocktail (Roche), loaded in SDS Sample Buffer [2× sample buffer was: 187.5 mm Tris–HCl (pH 6.8); 6% v/v SDS; 30% v/v glycerol; 0.03% w/v bromophenol blue (BioRad)] on 4–12% NuPage Bis‐Tris Gels under reducing conditions [5% beta‐mercaptoethanol (Sigma)] in MES buffer (Invitrogen) and transferred for 90 min at 300 mA to Hybond‐C Extra nitrocellulose membrane (Amersham Biosciences) using transfer buffer (25 mm Tris, 192 mm glycine, 25% methanol). Membranes were blocked in PBS, 5% Milk (Marvel semi‐skimmed) and 0.05% Tween‐20 (Sigma‐Aldrich). Incubation with primary antibody in blocking buffer was 1 h at room temperature. Membranes were washed 3× for 10 min in PBS‐Tween solution and incubated with HRP‐coupled secondary antibody in blocking buffer for 30–45 min at room temperature. Membranes were washed as before followed by an additional wash in PBS for 10 min. Blots were developed using ECL Western Blotting solutions (Amersham).

### Immunofluorescence microscopy and confocal imaging

CTL were washed into pre‐warmed serum‐free RPMI medium and allowed to settle onto hydrophobic multiwell slides (50 000 cells/well in 50 μL medium) for 20–25 min at 37°C. For conjugates P815 target cells were mixed at 1:1 ratio with human CTL , with 1 µg/mL PHA in RPMI at 4 × 10^6^ cells/mL concentration, incubated in suspension for 5 min, diluted to 1 × 10^6^ cells/mL, plated onto glass multiwell slides (Hendley), and incubated for a further 20–25 min at 37°C to adhere to the glass. Cells were fixed with ice‐cold methanol for 5 min on ice (except Figure 2C were cells were fixed in PFA for 20 min at room temperature), washed extensively in PBS prior to blocking in IF blocking buffer [1% BSA (Sigma‐Aldrich) in PBS] for 60 min at room temperature. All antibodies were diluted into IF blocking buffer. Samples were incubated with primary antibodies for 1 h at room temperature in a humidified environment, washed extensively and incubated with secondary antibody for 30–40 min in the dark. Samples were washed with blocking buffer and nuclei were stained with Hoechst diluted (1:25 000) in PBS for 7–10 min at room temperature. Samples were washed prior to mounting with non‐setting mounting medium (Vectashield, Vectalabs) and sealed with clear nail varnish.

Samples were imaged at room temperature with an Andor Revolution Spinning disk system with CSU‐X1 spinning disk (Yokogawa), 512 × 512, 16 µm^2^‐pixel camera (iXon, Andor) IX81 microscope (Olympus) using the 60× or 100× objective (numerical aperture 1.45) and 2.5× camera adapter and with lasers exciting at 405, 488 and 561 nm. Images were acquired using IQ2 (Andor) and processed using imaris software (Bitplane).

### Cytotoxicity assay

Cytotoxicity was determined with the CytoTox 96 Non‐Radioactive Cytotoxicity Assay (Promega) following manufacturers instructions. In brief: CTL were washed 2× in killing assay medium (RPMI 1640 medium minus phenol red, 2% fetal calf serum, FCS) and added in serial dilution to a round‐bottomed 96‐well plate to achieve titrated effector:target (E:T) ratios. P815 target cells were washed 2× and resuspended in killing assay media at 1 × 10^5^ cells/mL. Mouse anti‐human CD3ϵ antibody [UCHT‐1] (BD Biosciences, 555330) was added to the target cells at a concentration of 1 µg/mL (leading to a final concentration of 0.5 µg/mL upon addition of target cells to CTL suspension). Plates were incubated at 37°C. The absorbance of the supernatants at 490 nm determined the release of lactate dehydrogenase and percentage cytotoxicity after 2.5 h.

### Granule release assay

CTL of about 2–5 × 10^5^ were plated in 100 μL human CTL medium + 1.6 μL anti‐hLAMP1‐PE antibody per well on a flat bottom 96‐well plate (unstained and CD8+ single stained samples were plated in CTL medium only),100 μL human CTL medium containing 1 µg/mL Ionomycin plus 4 µm PMA or DMSO only were added to each well and the plate was incubated at 37°C. At each time point cells were transferred to a pre‐cooled round‐bottom 96‐well plate and spun down at 493 × ***g*** for 6 min at 5 °C. The medium was discarded, the cells were resuspended in 200 μL FACS buffer (PBS, 1% HI FCS, Biosera) per well and stored on ice in the dark until all time points were collected. Cells were pelleted, resuspended in FACS buffer plus anti‐CD8‐APC antibody (50 μL per well) and incubated for 30 min on ice in the dark. Cells were washed 1× and resuspended in 160 μL FACS buffer per well. Cells were analyzed using a FACSCalibur instrument (Becton Dickson) and flow jo software where gates were set as follows: Live cells (based on forward versus side scatter), CD8+ T‐cells (based on APC staining), CD8+/LAMP1+ T‐cells based on PE‐staining. The use of PE and APC fluorophores meant that no compensation was necessary during data acquisition. % of Max is ‘the number of cells in each bin divided by the number of cells in the bin that contains the largest number of cells’. Bins are defined as ‘numerical ranges for the parameter on the *X* axis’ (FlowJo manual, http://www.flowjo.com/v6/html/faq.html#2.4.4).
